# Clinicians’ Decision-Making Preferences Regarding Hypothetical Nanorobotic Applications in Head and Neck Tumors: A Vignette-Based Cross-Sectional Survey

**DOI:** 10.7759/cureus.109344

**Published:** 2026-05-21

**Authors:** Amandeep Kaur, Sasha Maria Menon, Amiya Kumar Nayak, Debadrita Ghosh, Samir Mansuri, Abdul Habeeb Bin Mohsin

**Affiliations:** 1 Department of Oral Health Sciences, Postgraduate Institute of Medical Education and Research Satellite Centre Sangrur, Sangrur, IND; 2 Department of Pediatric Anesthesiology, St. Jude Children's Research Hospital, Memphis, USA; 3 Department of Anesthesiology, Pandit Raghunath Murmu Medical College and Hospital, Baripada, IND; 4 Department of Pedodontics and Preventive Dentistry, Family Dental Clinic, Kolkata, IND; 5 Department of Oral and Maxillofacial Surgery, Al Kuwait Hospital, Dubai, ARE; 6 Department of Prosthodontics and Implantology, Coral Dental and Implants Centre, Hyderabad, IND

**Keywords:** head and neck cancer, nanorobotics, questionnaire, robotic surgical procedures, survey research

## Abstract

Introduction

Nanorobotic technology may represent a promising advancement in precision-based surgical interventions for head and neck tumors. This study aimed to evaluate clinicians’ decision-making preferences regarding hypothetical nanorobotic applications in the management of head and neck tumors using a vignette-based approach.

Materials and methods

This cross-sectional, vignette-based survey was conducted among oral and maxillofacial surgeons and anesthesiologists. A validated, self-administered questionnaire comprising demographic details and six clinical scenarios was used. Each vignette offered three treatment options: conventional, robot-assisted, and nanorobotic approaches, and assessed preference, confidence, perceived safety, willingness to adopt, and primary concerns. The data were then analyzed. Descriptive statistics were computed, and associations were evaluated using the chi-square test. Ordinal variables were analyzed using the Kruskal-Wallis test with Dunn’s post hoc correction.

Results

A total of 120 clinicians participated in the study. Nanorobotic approaches were significantly preferred in lymph node metastasis, deep-seated tumors, and medically compromised patients compared to conventional and robot-assisted approaches (p < 0.001), whereas conventional surgery was favored in facial nerve tumors (p = 0.012) and microvascular reconstruction (p = 0.008). No significant difference in treatment preference among conventional, robot-assisted, and nanorobotic approaches was observed in recurrent tumor scenarios (p = 0.843). The mean confidence and perceived safety scores for the nanorobotic approach were highest in deep-seated tumors (3.9 ± 0.8 and 3.8 ± 0.7, respectively) and lowest in reconstructive scenarios (3.1 ± 1.0 and 2.9 ± 1.1, respectively). Across all vignette responses, 49.7% indicated willingness to adopt nanorobotic interventions. Prior robotic surgery experience showed a significant association with preference for nanorobotics (p = 0.039). Cost, lack of evidence, training, and safety-related concerns were commonly reported barriers to nanorobotic adoption, and nanotechnology familiarity significantly influenced confidence and perceived safety (p < 0.001).

Conclusion

Clinician acceptance of nanorobotic interventions is scenario-dependent and influenced by prior technological exposure, with safety, control, cost, lack of evidence, and training requirements remaining important concerns and barriers to adoption. While nanorobotic approaches show promise in precision-driven and minimally invasive scenarios, their integration into routine clinical practice will require robust evidence, structured training, and technological refinement. Clinically, these findings suggest that early adoption may be most feasible in selected cases requiring high precision and limited access, and that targeted clinician education and simulation-based training could facilitate smoother translation into practice.

## Introduction

Head and neck tumors represent a diverse group of neoplasms that often involve anatomically complex regions in close proximity to critical neurovascular structures. Surgical management remains an important cornerstone of treatment; however, it is frequently associated with significant morbidity, functional impairment, and esthetic challenges [1]. Advances in surgical technology, particularly robotic-assisted systems, have improved precision and minimally invasive capabilities, yet limitations such as restricted accessibility, high cost, and dependence on operator skill persist.

Nanotechnology has emerged as a promising frontier in medicine, offering potential applications in targeted drug delivery, diagnostics, and minimally invasive therapeutic interventions. Nanorobotics, a conceptual extension of nanotechnology, proposes the use of nanoscale devices capable of navigating biological environments with high precision [2,3]. In the context of head and neck oncology, nanorobotic interventions could theoretically enable targeted tumor removal, improve preservation of vital structures, and reduce postoperative complications [4]. Despite these potential advantages, nanorobotic surgery remains largely hypothetical, and its clinical adoption depends heavily on clinician acceptance, perceived safety, and feasibility.

Clinical decision-making in surgery is influenced by multiple factors, including practitioner experience, familiarity with emerging technologies, perceived risks, and patient-related considerations. Vignette-based studies provide a useful method for simulating real-world clinical scenarios and assessing decision-making behavior in a controlled manner [5]. Understanding clinician preferences and concerns regarding nanorobotic interventions is essential for identifying barriers to adoption and guiding future research and technological development.

This study aimed to evaluate clinicians’ decision-making preferences regarding the use of hypothetical nanorobotic interventions in the management of head and neck tumors using a vignette-based approach. The primary objective of this vignette-based study was to evaluate clinicians’ preferred treatment modality among conventional surgery, robotic-assisted surgery, and hypothetical nanorobotic interventions across different head and neck oncologic scenarios. The secondary objectives were to assess clinicians’ confidence levels, perceived safety, and willingness to adopt nanorobotic approaches in clinical practice, as well as to identify factors influencing decision-making and acceptance, including prior exposure to advanced technologies, familiarity with nanotechnology, and perceived barriers such as safety, control, cost, and training.

## Materials and methods

Study design and setting

This cross-sectional, vignette-based questionnaire study was coordinated by the Department of Anesthesiology at Pandit Raghunath Murmu Medical College and Hospital, Baripada, India. The study was conducted over a period of six months, from February 2025 to July 2025, with data collection carried out across multiple tertiary care hospitals, dental institutions, and private clinical settings to ensure diversity in clinical exposure and practice patterns. Ethical approval was obtained from the institutional ethics committee (IEC/426 dated 01-01-2025).

Study population

The study population comprised clinicians from the specialties of oral and maxillofacial surgery and anesthesiology. Participants were included if they had a minimum of one year of post-qualification clinical experience and were actively involved in the surgical or perioperative management of head and neck cases. Clinicians who were not involved in such procedures or were unwilling to provide consent were excluded. A non-probability convenience sampling technique was used, and participants were recruited through institutional mailing lists, professional WhatsApp groups, and direct communication.

Sample size calculation

Sample size estimation was performed using G*Power software (version 3.1.9.7; Heinrich Heine University Düsseldorf). Based on an anticipated acceptance proportion of approximately 55%, derived from prior literature on healthcare professionals’ acceptance of emerging medical technologies [6], a minimum sample size of 108 participants was calculated to achieve 80% statistical power at a two-sided significance level of α = 0.05, assuming a medium effect size of 0.30. To account for a potential non-response or incomplete data rate of approximately 10%, the final sample size was increased to 120 participants.

Questionnaire development

A structured, self-administered questionnaire was developed following an extensive review of the literature on nanotechnology, robotic surgery, and clinical decision-making models. The questionnaire consisted of three sections: demographic and professional characteristics, vignette-based clinical scenarios, and decision-making assessment. The demographic section included variables such as age, sex, specialty, years of experience, type of practice, exposure to robotic surgery, and familiarity with nanotechnology (Appendix 1). The core component comprised six clinical vignettes representing complex head and neck oncological scenarios, including tumors near critical neurovascular structures, lymph node metastasis, deep-seated tumors, reconstructive procedures, medically compromised patients, and recurrent tumors (Appendix 2). Each vignette provided three treatment options: conventional surgery, robot-assisted surgery, and a hypothetical nanorobotic approach. Participants were asked to select their preferred modality and respond to follow-up questions assessing confidence, perceived safety, willingness to adopt, and primary concerns. General perception items regarding nanorobotic interventions and barriers to adoption were also included in the questionnaire (Appendix 3). The nanorobotic option was presented uniformly across all vignettes as a hypothetical advanced minimally invasive technology intended for highly precise targeted intervention in anatomically complex regions. All participants received the vignettes in the same predefined sequence to maintain consistency in scenario interpretation and response flow.

Questionnaire validation

Content validation of the questionnaire was performed by a panel of six experts, including three oral surgeons and three anesthesiologists. The item-level content validity index ranged from 0.83 to 1.00, whereas the scale-level content validity index (S-CVI/Ave) was 0.92. The content validity ratio ranged from 0.78 to 1.00, confirming the relevance and essentiality of all items. A pilot study was conducted among 20 clinicians to assess clarity and feasibility, and minor modifications were made. Internal consistency was evaluated using Cronbach’s alpha, which was 0.88, with subscale reliability values of 0.85 for decision-making items and 0.87 for perception-related items, indicating good reliability.

Questionnaire distribution and assessment

The final questionnaire was converted into a digital format using Google Forms and distributed electronically. Participation was voluntary, and informed consent was obtained before participation. Responses were collected anonymously to ensure confidentiality and minimize response bias, and duplicate entries were restricted. The primary outcome measure was the preferred treatment modality in each vignette. Secondary outcomes included confidence level, perceived safety, willingness to adopt nanorobotic technology, and primary concerns, such as safety, cost, training, and control.

Statistical analysis

All data were entered into Microsoft Excel and analyzed using IBM SPSS Statistics (version 26.0; IBM Corp., Armonk, NY, USA). Descriptive statistics were used to summarize the data, with categorical variables expressed as frequencies and percentages and continuous variables expressed as mean ± SD. The chi-square test was used to assess associations between categorical variables, including preferred surgical approaches across vignettes and respondent characteristics. Because confidence level and perceived safety were ordinal variables, they were analyzed using the Kruskal-Wallis test, followed by post hoc Dunn’s test with Bonferroni correction for pairwise comparisons. Statistical significance was set at p < 0.05. Statistical analyses were performed separately for each vignette scenario rather than on pooled independent observations.

## Results

Approximately 165 clinicians were approached, of whom 120 completed the survey, yielding a response rate of 72.7%. The demographic and professional characteristics of the respondents are summarized in Table 1. The sample predominantly consisted of experienced clinicians, with the majority being oral and maxillofacial surgeons actively involved in head and neck case management. Exposure to robotic surgery was nearly balanced, whereas familiarity with nanotechnology was generally low to moderate.

**Table 1:** Demographic and professional characteristics of the respondents (N = 120). Values are expressed as frequencies (n) and percentages (%). Percentages may not total 100% because of rounding.

Variable	Category	N = 120	%
Age (years)	< 30	8	6.70%
	30-39	30	25.00%
	40-49	42	35.00%
	50-59	28	23.30%
	≥ 60	12	10.00%
Sex	Male	74	61.70%
	Female	46	38.30%
Specialty	Oral and maxillofacial surgery	68	56.70%
	Anesthesiology	52	43.30%
Years of experience	< 5 years	18	15.00%
	5-10 years	32	26.70%
	10-20 years	45	37.50%
	> 20 years	25	20.80%
Type of practice	Academic	48	40.00%
	Private	38	31.70%
	Both	34	28.30%
Involvement in head and neck cases	Yes	98	81.70%
	No	22	18.30%
Robotic surgery experience	Yes	64	53.30%
	No	56	46.70%
Nanotechnology familiarity	Low	42	35.00%
	Moderate	55	45.80%
	High	23	19.20%

The distribution of preferred treatment modalities across the six clinical vignettes is presented in Table 2. A statistically significant variation in treatment preference was observed across most scenarios. Nanorobotic approaches were more frequently favored in situations requiring high precision or limited access, whereas conventional approaches were preferred in scenarios involving a higher risk to critical structures. No significant difference in preference was observed in the recurrent tumor scenario, indicating relative clinical equipoise.

**Table 2:** Preferred surgical approaches across clinical vignettes. Values are presented as frequencies (n) and percentages (%). The chi-square test was used to compare the frequency distribution of the three approaches within each vignette. *Significant at p < 0.05.

Clinical vignette	Conventional n (%)	Robot-assisted n (%)	Nanorobotic n (%)	Chi-square statistic	p-value
V1: Facial nerve tumor (paralysis risk)	52 (43.3)	38 (31.7)	30 (25.0)	6.2	0.012*
V2: Lymph node metastasis (precision)	20 (16.7)	34 (28.3)	66 (55.0)	27.8	< 0.001*
V3: Deep-seated tumor (limited access)	18 (15.0)	32 (26.7)	70 (58.3)	36.2	< 0.001*
V4: Microvascular reconstruction	58 (48.3)	30 (25.0)	32 (26.7)	12.2	0.008*
V5: Medically compromised patient	22 (18.3)	28 (23.3)	70 (58.3)	34.31	< 0.001*
V6: Recurrent tumor near major vessels	42 (35.0)	40 (33.3)	38 (31.7)	0.2	0.843

Confidence levels, perceived safety ratings, and willingness to adopt nanorobotic technology are summarized in Table 3. Overall, higher confidence and perceived safety scores were observed in scenarios involving deep-seated tumors and lymph node metastasis, whereas lower scores were noted in reconstructive contexts. Willingness to adopt nanorobotic interventions varied across scenarios, reflecting context-dependent acceptance.

**Table 3:** Clinician confidence, perceived safety, and willingness to adopt nanorobotic interventions across clinical vignettes. Confidence = clinician confidence in selecting the preferred approach; perceived safety = clinician-perceived safety of nanorobotics in the described scenario.

Vignette	Confidence (mean ± SD)	Perceived safety (mean ± SD)	Willingness to adopt: yes n (%)	Primary concern
V1: Facial nerve tumor	3.2 ± 0.9	3.0 ± 1.0	42 (35.0)	Safety
V2: Lymph node metastasis	3.8 ± 0.7	3.6 ± 0.8	72 (60.0)	Training
V3: Deep-seated tumor	3.9 ± 0.8	3.8 ± 0.7	78 (65.0)	Control
V4: Microvascular reconstruction	3.1 ± 1.0	2.9 ± 1.1	40 (33.3)	Safety
V5: Medically compromised patient	3.7 ± 0.7	3.6 ± 0.8	76 (63.3)	Cost
V6: Recurrent tumor	3.4 ± 0.9	3.2 ± 1.0	50 (41.7)	Safety
Overall (pooled)	3.52 ± 0.83	3.35 ± 0.90	358/720 (49.7)	-

The association between clinician characteristics and preference for nanorobotic approaches is shown in Table 4. Among the variables assessed, prior experience with robotic surgery demonstrated a statistically significant association with preference for nanorobotic interventions, whereas years of experience and familiarity with nanotechnology did not show significant associations.

**Table 4:** Association between respondent characteristics and preference for nanorobotic interventions. Values are presented as frequencies (n) and percentages (%). χ² = chi-square statistic. *Statistically significant at p < 0.05.

Variable	Category	Preferred nanorobotics n (%)	Other n (%)	χ²	P-value
Years of experience	<5 years	12 (66.7)	6 (33.3)	5.82	0.121
	5-10 years	18 (56.3)	14 (43.8)		
	10-20 years	22 (48.9)	23 (51.1)		
	> 20 years	10 (40.0)	15 (60.0)		
Robotic surgery experience	Yes	38 (59.4)	26 (40.6)	4.27	0.039*
	No	24 (42.9)	32 (57.1)		
Nanotechnology familiarity	Low	18 (42.9)	24 (57.1)	3.41	0.181
	Moderate	30 (54.5)	25 (45.5)		
	High	14 (60.9)	9 (39.1)		

The distribution of primary concerns influencing decision-making within each clinical vignette is detailed in Table 5. Safety and control emerged as the most frequently reported vignette-based concerns, with variations depending on the clinical context. Cost and training were also identified as relevant but less dominant factors.

**Table 5:** Distribution of primary concerns across clinical vignettes. Each respondent selected one primary concern per vignette: safety, cost, training, or control. The total pooled sample included 720 responses from 120 respondents across six vignettes. Values are presented as frequencies (n) and percentages (%).

Vignette	Safety n (%)	Cost n (%)	Training n (%)	Control n (%)	Total n (%)
V1: Facial nerve tumor	42 (35.0)	24 (20.0)	18 (15.0)	36 (30.0)	120 (100.0)
V2: Lymph node metastasis	20 (16.7)	22 (18.3)	38 (31.7)	40 (33.3)	120 (100.0)
V3: Deep-seated tumor	18 (15.0)	16 (13.3)	36 (30.0)	50 (41.7)	120 (100.0)
V4: Microvascular reconstruction	48 (40.0)	34 (28.3)	24 (20.0)	14 (11.7)	120 (100.0)
V5: Medically compromised patient	28 (23.3)	44 (36.7)	22 (18.3)	26 (21.7)	120 (100.0)
V6: Recurrent tumor	42 (35.0)	22 (18.3)	12 (10.0)	44 (36.7)	120 (100.0)
Total (pooled)	198 (27.5)	162 (22.5)	150 (20.8)	210 (29.2)	720 (100.0)

Further analysis using the Kruskal-Wallis test demonstrated a statistically significant association between levels of nanotechnology familiarity and both confidence and perceived safety (Table 6). Post hoc analysis using Dunn’s test with Bonferroni correction confirmed significant pairwise differences, particularly between clinicians with low and high familiarity (Table 7). Overall, the findings indicate that clinician preference for nanorobotic interventions is influenced by the clinical scenario, prior exposure to advanced technologies, and perceived safety and control considerations.

**Table 6:** Association of nanotechnology familiarity with confidence and perceived safety using the Kruskal-Wallis test. df: Degrees of freedom. The Kruskal-Wallis test was used to compare groups. *Statistically significant at p < 0.05.

Outcome variable	Low familiarity (n = 42), median (IQR)	Moderate familiarity (n = 55), median (IQR)	High familiarity (n = 23), median (IQR)	H statistic	df	p-value
Confidence level (1-5)	3.0 (2.5-3.5)	3.5 (3.0-4.0)	4.0 (3.5-4.5)	34.72	2	< 0.001*
Perceived safety (1-5)	2.8 (2.3-3.3)	3.3 (2.8-3.8)	3.8 (3.3-4.3)	26.2	2	< 0.001*

**Table 7:** Post hoc pairwise comparisons of clinician confidence and perceived safety across levels of nanotechnology familiarity using Dunn’s test with Bonferroni correction. Dunn’s test was performed for pairwise comparisons following a significant Kruskal-Wallis test. Bonferroni correction was applied for multiple comparisons. *Statistically significant at p < 0.05 after adjustment.

Comparison	Confidence level z statistic	Confidence level p-value (unadjusted)	Confidence level p-value (Bonferroni)	Perceived safety z statistic	Perceived safety p-value (unadjusted)	Perceived safety p-value (Bonferroni)
Low vs. Moderate	-2.996	0.003*	0.008*	-2.103	0.036*	0.107
Low vs. High	-5.878	< 0.001*	< 0.001*	-5.114	< 0.001*	< 0.001*
Moderate vs. High	-3.669	< 0.001*	< 0.001*	-3.608	< 0.001*	< 0.001*

**Figure 1:**
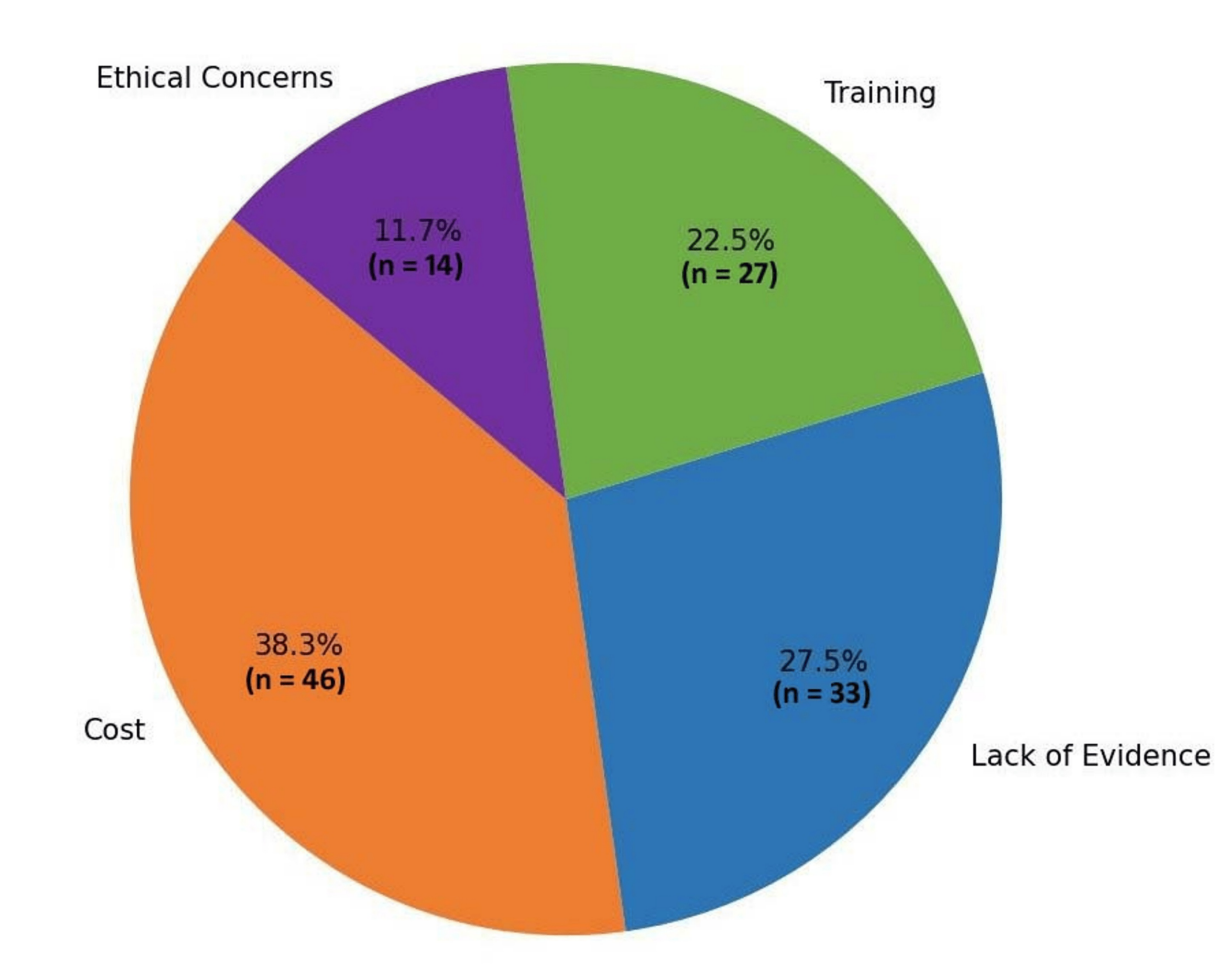
Distribution of overall perceived barriers influencing clinician decision-making regarding nanorobotic adoption. Figure represents responses to the general barrier assessment item evaluating overall perceived barriers toward nanorobotic adoption, including cost, lack of evidence, training requirements, and ethical concerns. Values are presented as percentages of total respondents (n = 120).

Figure 1 illustrates the distribution of overall perceived barriers influencing clinicians’ decision-making regarding nanorobotic adoption. This figure represents responses to a separate general barrier assessment item and is distinct from the vignette-based concerns summarized in Table 5. Cost was the most commonly reported overall barrier, followed by lack of evidence, training requirements, and ethical concerns.

## Discussion

The present study explored clinicians’ decision-making preferences regarding the hypothetical use of nanorobotic interventions for head and neck tumors using a vignette-based approach. The findings demonstrate a clear pattern of scenario-dependent acceptance, with nanorobotic approaches favored in conditions requiring high precision and minimally invasive access, whereas conventional techniques remain preferred in anatomically sensitive or reconstructive scenarios. These results highlight the cautious yet optimistic attitude of clinicians toward emerging nanotechnologies.

A key observation of this study was the significantly higher preference for nanorobotic interventions in scenarios such as lymph node metastasis, deep-seated tumors, and medically compromised patients. These clinical situations often demand enhanced precision, minimal collateral tissue damage, and improved accessibility, which are attributes theoretically offered by nanorobotic systems. This aligns with previous literature suggesting that nanotechnology-based interventions have the potential to revolutionize targeted therapy and minimally invasive procedures [7,8]. Similarly, advances in robotic surgery have demonstrated improved surgical precision and reduced morbidity in head and neck oncology, supporting the translational potential of nanorobotic concepts [9]. Furthermore, the success of robotic surgery in head and neck oncology has demonstrated improved precision and reduced morbidity, supporting the translational relevance of nanorobotic concepts [10].

By contrast, conventional surgery remained the preferred approach in scenarios involving facial nerve proximity and microvascular reconstruction. These findings likely reflect clinicians’ concerns regarding safety, tactile feedback, and procedural control, which are critical in surgeries involving delicate neurovascular structures. Despite their advantages, robotic systems still face limitations in haptic feedback and intraoperative adaptability, and these limitations may be perceived as even more pronounced in the context of nanorobotics, which remains largely theoretical [11]. The absence of a dominant preference in the recurrent tumor scenario suggests clinical equipoise, where uncertainty and procedural complexity influence reliance on established surgical methods. This aligns with existing evidence indicating that surgeons tend to favor familiar approaches in high-risk or uncertain clinical situations [12].

An important observation of this study was that prior robotic surgery experience significantly influenced preference for nanorobotic interventions, whereas years of experience and familiarity with nanotechnology did not show independent associations. This highlights the role of hands-on exposure and technological familiarity in shaping clinician acceptance. Supporting this, large clinical series have demonstrated that increased experience with robotic systems improves surgical confidence, reduces the learning curve, and enhances the adoption of advanced techniques [6]. These findings suggest that familiarity with existing robotic platforms may serve as a stepping stone for future nanorobotic integration.

With respect to perception-based outcomes, higher confidence levels and perceived safety ratings were observed in precision-oriented scenarios, whereas lower values were observed in reconstructive contexts. Furthermore, the significant association between nanotechnology familiarity and both confidence and perceived safety suggests that education and awareness play crucial roles in technology acceptance. Similar trends have been reported in previous literature evaluating emerging medical technologies, where familiarity is directly linked to increased acceptance and reduced perceived risk [13,14].

The analysis of vignette-based concerns revealed that control and safety were the most prominent concerns, followed by cost and training. In contrast, the separate overall barrier assessment showed that cost was the most commonly reported barrier to nanorobotic adoption, followed by lack of evidence, training requirements, and ethical concerns. These findings suggest that clinicians prioritize procedural reliability and patient outcomes when evaluating scenario-specific use, while broader adoption may also be influenced by financial, evidentiary, and training-related challenges.

Overall, the findings of this study suggest that while nanorobotic interventions are viewed favorably in specific clinical contexts, their widespread adoption depends on addressing key concerns related to safety, control, and real-world feasibility. The vignette-based methodology provides valuable insights into clinician reasoning; however, actual clinical behavior may differ when such technologies become available [15-16].

Clinical implications and limitations

The findings of this study provide preliminary insight into clinician perceptions and decision-making preferences regarding hypothetical nanorobotic applications in head and neck oncology. The results suggest that clinicians may demonstrate greater acceptance of advanced minimally invasive technologies in precision-oriented clinical scenarios, which may help guide future research, technology development, and educational initiatives related to emerging surgical innovations. However, several limitations should be considered when interpreting the findings. The study was based on hypothetical vignette scenarios and therefore may not fully represent real-world clinical behavior or actual treatment decisions. Since nanorobotic interventions are not yet clinically available, participant responses were dependent on their conceptual understanding and perceived feasibility of the described technology. The use of convenience sampling and recruitment from a limited geographic setting may reduce the generalizability of the findings to broader clinician populations. In addition, the study relied on self-reported responses, which may be influenced by perception and response bias. Although all participants received standardized vignettes and identical descriptions of treatment options, scenario-based responses may still vary according to individual interpretation and prior technological exposure. Furthermore, statistical analyses were performed separately for each vignette scenario, and these findings should therefore be interpreted within the exploratory framework of a cross-sectional, vignette-based survey. Future multicenter studies with larger and more diverse samples, along with eventual real-world clinical validation, are needed to further evaluate clinician acceptance and the practical applicability of such technologies.

## Conclusions

This study demonstrated that clinicians showed selective acceptance of nanorobotic interventions in head and neck oncology, favoring their use in precision-driven and minimally invasive scenarios, such as deep-seated tumors and metastatic conditions. However, conventional approaches remained preferred in anatomically complex and high-risk procedures owing to concerns regarding safety and procedural control. Prior experience with robotic surgery significantly influenced acceptance, highlighting the role of technological exposure. Key concerns and barriers, particularly safety, control, cost, lack of evidence, and training requirements, must be addressed before clinical application. Overall, nanorobotics hold promising potential; however, their successful integration depends on robust evidence, clinician training, and technological advancements that ensure reliability and patient safety.

## Appendices

Appendix 1

**Table 8:** Demographic and professional details of the respondents. The questionnaire used in this study was developed by the study authors.

Variable	Response options
Age (years)	-
Sex	Male / Female / Prefer not to say
Specialty	Oral and maxillofacial surgery / Anesthesiology / Other
Years of clinical experience	<5 years / 5-10 years / 10-20 years / >20 years
Type of practice	Academic / Private / Both
Involvement in head and neck surgical cases	Yes / No
Prior exposure to robot-assisted surgery	Yes / No
Familiarity with nanotechnology in medicine	Low / Moderate / High

Appendix 2

**Table 9:** Standardized clinical vignettes used to assess clinicians’ decision-making preferences regarding nanorobotic interventions in head and neck tumors. For each scenario, participants were asked to select the most appropriate option and answer the related questions. The questionnaire used in this study was developed by the study authors.

Vignette	Clinical scenario	Preferred approach	Confidence (1-5)	Perceived safety (1-5)	Willingness to adopt (Yes/No)	Primary concern
1	A patient presents with a benign or malignant tumor located in close proximity to the facial nerve, where surgical excision carries a significant risk of nerve injury and potential postoperative facial paralysis. Precise tumor removal with maximal preservation of nerve function is critical.	Conventional / Robot-assisted / Nanorobotic	-	-	Yes / No	Safety / Cost / Training / Control
2	A patient presents with limited lymph node metastasis in the head and neck region, where accurate localization and targeted removal of metastatic nodes are essential to achieve optimal oncologic control while minimizing damage to surrounding healthy tissues.	Conventional / Robot-assisted / Nanorobotic	-	-	Yes / No	Safety / Cost / Training / Control
3	A patient presents with a deep-seated tumor located in an anatomically complex region with limited surgical access, where achieving adequate exposure is challenging and precise tumor removal is required while minimizing damage to adjacent vital structures.	Conventional / Robot-assisted / Nanorobotic	-	-	Yes / No	Safety / Cost / Training / Control
4	A patient requires surgical resection of a head and neck tumor followed by microvascular reconstruction, where precise tissue handling and accurate vascular anastomosis are critical to ensure flap viability and optimal functional and esthetic outcomes.	Conventional / Robot-assisted / Nanorobotic	-	-	Yes / No	Safety / Cost / Training / Control
5	A medically compromised patient with multiple systemic comorbidities requires surgical management of a head and neck tumor, where minimizing surgical trauma, operative time, and postoperative complications is essential to reduce overall risk and improve recovery.	Conventional / Robot-assisted / Nanorobotic	-	-	Yes / No	Safety / Cost / Training / Control
6	A patient presents with a recurrent head and neck tumor located in close proximity to major blood vessels, where previous treatment has altered the anatomy, increasing surgical complexity and the risk of intraoperative vascular injury and complications.	Conventional / Robot-assisted / Nanorobotic	-	-	Yes / No	Safety / Cost / Training / Control

Appendix 3

**Table 10:** General perceptions regarding nanorobotic interventions. The questionnaire used in this study was developed by the study authors.

Question	Response options
Nanorobotic surgery represents the future of surgical care	Agree / Neutral / Disagree
Willingness to undergo training in nanorobotic surgery	Yes / No
Belief that nanorobotics will improve patient outcomes	Yes / No
Major barrier to adoption	Cost / Lack of evidence / Training / Ethical concerns
